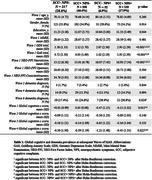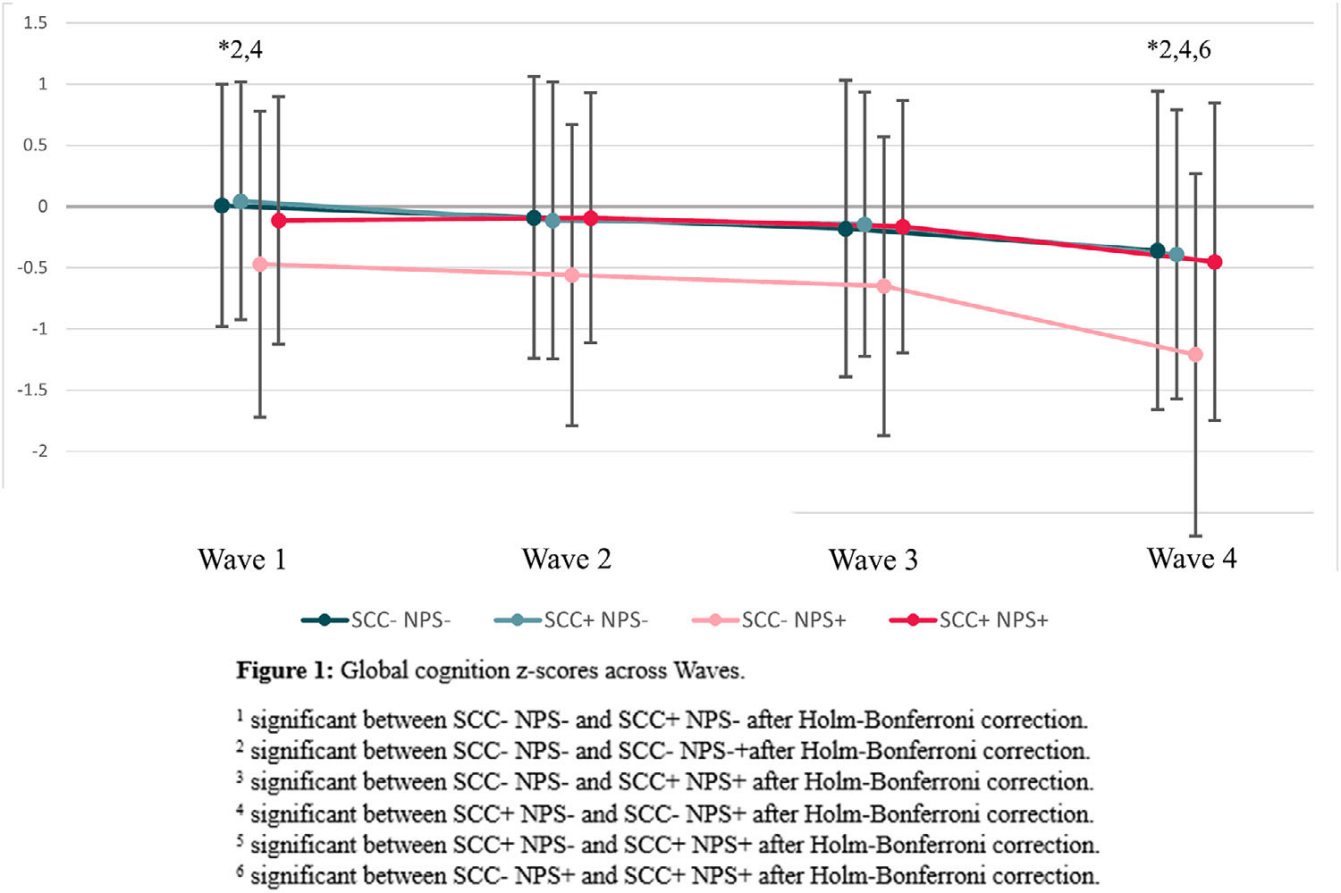# Exploring relationships among neuropsychiatric symptoms, subjective cognitive complaints, cognitive performance, and incident dementia: Findings from the Sydney Memory and Ageing Study

**DOI:** 10.1002/alz.092087

**Published:** 2025-01-03

**Authors:** Katya T. Numbers, Russell J Chander, Jason Chen, Henry Brodaty

**Affiliations:** ^1^ Centre for Healthy Brain Ageing (CHeBA), University of New South Wales, UNSW Sydney, NSW Australia

## Abstract

**Background:**

Subjective cognitive complaints (SCCs) and neuropsychiatric symptoms (NPS) are emerging as potential early indicators of neurodegenerative diseases like Alzheimer’s disease (AD). SCCs refers to a self‐perceived decline in cognitive abilities without objective impairment, while NPS describe neuropsychiatric symptoms that emerge in later life that may precede or co‐occur with cognitive decline. This study explores the association between SCCs, NPS, global cognition, and incident dementia using data from the Sydney Memory and Ageing Study (MAS).

**Method:**

Participants were 754 older adults from MAS (M age = 78.76, SD = 4.71) with a diagnosis of normal cognition at baseline. SCC was determined based on participants reporting having noticed changes in their memory. NPS was defined as having scored >1 on the Neuropsychiatric Inventory (NPI) total score. Participants were categorised into four groups based on the presence of SCC and/or NPS. Outcome variables were global cognition (composite z‐score derived from tests of attention/processing speed, language, executive functions, visuospatial abilities, and memory) and clinical consensus diagnosis of dementia according to DSM‐IV criteria.

**Result:**

Of the total participants, 217 (28.8%) had neither SCC nor NPS (SCC‐ NPS‐), 350 (46.4%) had SCC only (SCC+ NPS‐), 52 (6.9%) had NPS only (SCC‐ NPS+), and 236 had both SCC and NPS (NPS+SCC+). At baseline, SCC‐ NPS+ had significantly poorer global cognition than SCC‐ NPS‐ or SCC+ NPS‐. At wave 4 (6‐year follow‐up), SCC‐ NPS+ showed a steeper decline in global cognition compared to other groups. A greater proportion of SCC+ NPS+ at Wave 6 (10‐year follow‐up) had developed dementia compared to SCC‐ NPS‐. Multivariable logistic regression with age at baseline as the only covariate surviving stepwise elimination showed SCC‐ NPS+ (OR 3.70; SE 2.15; p = 0.025) and SCC+ NPS+ (OR 3.18; SE 1.25; p = 0.003) had increased odds over SCC‐ NPS‐ in developing dementia by Wave 6.

**Conclusion:**

NPS is associated with poorer global cognition and, with or without SCC, is a significant long‐term risk factor for dementia. The findings highlight the potential importance of considering NPS alongside cognitive features, and suggest that assessing both neurobehavioral and neurocognitive dimensions could offer a more comprehensive risk assessment in cognitively normal individuals.